# Bacteria dialog with Santa Rosalia: Are aggregations of cosmopolitan bacteria mainly explained by habitat filtering or by ecological interactions?

**DOI:** 10.1186/s12866-014-0284-5

**Published:** 2014-12-04

**Authors:** Alberto Pascual-García, Javier Tamames, Ugo Bastolla

**Affiliations:** Centro de Biología Molecular Severo Ochoa (CSIC-UAM), c. Nicolás Cabrera 1, campus UAM, Madrid, E-28049 Spain; Centro Nacional de Biotecnologí a (CSIC) c. Darwin 3, campus UAM, Madrid, E-28049 Spain

**Keywords:** Bacterial ecology, Habitat filtering, Biodiversity, Cooperation, Syntrophy, Bacterial speciation, Black queen hypothesis, Ecological null-models, Ecological networks

## Abstract

**Background:**

Since the landmark Santa Rosalia paper by Hutchinson, niche theory addresses the determinants of biodiversity in terms of both environmental and biological aspects. Disentangling the role of habitat filtering and interactions with other species is critical for understanding microbial ecology. Macroscopic biogeography explores hypothetical ecological interactions through the analysis of species associations. These methods have started to be incorporated into microbial ecology relatively recently, due to the inherent experimental difficulties and the coarse grained nature of the data.

**Results:**

Here we investigate the influence of environmental preferences and ecological interactions in the tendency of bacterial taxa to either aggregate or segregate, using a comprehensive dataset of bacterial taxa observed in a wide variety of environments. We assess significance of taxa associations through a null model that takes into account habitat preferences and the global distribution of taxa across samples. The analysis of these associations reveals a surprisingly large number of significant aggregations between taxa, with a marked community structure and a strong propensity to aggregate for cosmopolitan taxa. Due to the coarse grained nature of our data we cannot conclusively reject the hypothesis that many of these aggregations are due to environmental preferences that the null model fails to reproduce. Nevertheless, some observations are better explained by ecological interactions than by habitat filtering. In particular, most pairs of aggregating taxa co-occur in very different environments, which makes it unlikely that these associations are due to habitat preferences, and many are formed by cosmopolitan taxa without well defined habitat preferences. Moreover, known cooperative interactions are retrieved as aggregating pairs of taxa. As observed in similar studies, we also found that phylogenetically related taxa are much more prone to aggregate than to segregate, an observation that may play a role in bacterial speciation.

**Conclusions:**

We hope that these results stimulate experimental verification of the putative cooperative interactions between cosmopolitan bacteria, and we suggest several groups of aggregated cosmopolitan bacteria that are interesting candidates for such an investigation.

**Electronic supplementary material:**

The online version of this article (doi:10.1186/s12866-014-0284-5) contains supplementary material, which is available to authorized users.

## Background

In his seminal paper *Homage to Santa Rosalia* or *Why are there so many kinds of animals?* [1], George E. Hutchinson addressed the determinants of biodiversity in light of a renewed concept of niche. Hutchinson’s question has an interesting challenge in the microbial world. Understanding the determinants of bacterial niches can open new perspectives on plant and animal evolution as well, since bacteria co-evolved with multicellular eukaryotes for hundreds of millions of years mutually influencing each other [2], and it may have important biomedical applications. An increasing quantity of data from high-throughput experiments is now available for large scale ecological studies of bacterial communities, in particular for the human microbiome [3-6]. Early analysis suggested that ecological patterns are qualitatively similar for macro- and microorganisms [7] and allowed identifying taxa-area and distance decay relationships [8,9] and the influence of environmental variables such as depth [10] or salinity [11], stimulating the emergence of prokaryotic biogeography [12,13].

Data on presence or absence of species in different locations are used by biogeographists to infer ecological processes [14]. Similarly, presence-absence matrices obtained by sequencing environmental samples or by mining abstracts of scientific papers [15] offer new opportunities to shed light son bacterial ecology. Recently, several groups used large-scale data to study bacterial associations [16-24], reviewed in [25]. In the present work, we analyse bacterial species associations for a comprehensive collection of samples from a large variety of environments classified at the three hierarchical levels of environmental subtype, type and supertype [26]. We assess the significance of their associations by means of a recently proposed null-model [27] that optimally reproduces the global distribution of taxa across samples, and that we modified to take into account environmental preferences. To this end, we exploited the hierarchical classification of samples into environmental groups developed by Tamames et al. [26] (see Additional file 1: Figure S1 and Table S1) and developed a new analytical pairwise score.

We are interested in pairs of taxa that aggregate and segregate, which means that they co-occurr significantly more often and less often than expected based on the null model. Aggregations and segregations can be attributed to habitat preferences, to direct ecological interactions (cooperative interactions, commensalism and parasitism for aggregation, competitive interactions for segregation) or to indirect interactions with another species or group of species. We consider environmental preferences in our null model, with the aim to reduce the number of aggregations that are due to common preferences. We try to estimate how many aggregations may be due to environmental preferences that are not removed by the null model by differentiating associations that occur in a specific environment from those that are not specific. Another way to perform this analysis consists in focusing on cosmopolitan taxa that do not show apparent environmental preferences but are found in many diverse environments. The previous work of Tamames et al. [26] found that, at the genus level that we consider here, cosmopolitanism is not rare among bacteria. We aim at investigating in which way ecological associations may contribute to this property.

In this work, we assess all possible segregations and aggregations of 1187 bacterial taxa corresponding to the genus level, observed in 2322 samples from different environments, and we analyse the relationship between these environmental associations on the one hand and cosmopolitanism and known ecological associations on the other hand.

## Results

### Constructing networks of bacterial associations

Our first aim is to construct a null model that optimally represents the global distribution of taxa across samples, considering their habitat preferences at the level of environmental subtypes, but assuming that taxa do not interact between themselves. This approach is different from most current approaches to microbial community studies in that it explicitly considers habitat preferences, and in that the association score of a pair of taxa depends on the observed distribution of all other taxa.

Since for many samples abundance information is not present, the 2322 samples were transformed into the binary presence-absence matrix *X*_*ia*_∈{0,1}, where *i* labels one of the *N* taxa and *a* labels one of the *M* samples. To limit the bias caused by the choice of primers in sequencing experiments, we excluded experiments targeted at detecting specific taxa (see Methods). We adopt the probabilistic null model proposed by Navarro-Alberto and Manly [27], in which the probability *π*_*ia*_ that taxon *i* is observed at sample *a* in the absence of taxa interactions is parametrized as *π*_*ia*_=1− exp(−*p*_*i*_*q*_*a*_) where the parameter *p*_*i*_ is related with the abundance of taxon *i* and *q*_*a*_ is related with the biodiversity supported by sample *a*, respectively.

The *M*+*N* parameters *p*_*i*_ and *q*_*a*_ are determined by maximum likelihood, so that the resulting null model is most difficult to reject. We take into account that each taxon has a preference for some habitat by assuming that the taxa parameters *p*_*i*_(*A*) are specific for each environmental subtype *A* (see Methods). If taxon *i* is never found in environment *A*, then *p*_*i*_(*A*)=0, implying that *π*_*ia*_=0 if *a*∈*A*, i.e. the taxon is never found in samples of environment *A* simulated through the null model either.

The significance of the observed co-occurrences is analytically assessed through the *aggregation score*$S_{\textit {ij}}^{\mathrm {A}}\,=\,-\log \mathrm {P}_{\textit {ij}}\left (n\geq n_{\textit {ij}}\right)$, where *n*_*ij*_ is the observed number of samples where taxa *i* and *j* co-occur and P_*ij*_(*n*) is the null-model probability that taxa *i* and *j* co-occur at *n* locations (see Association scores in Methods). Similarly, we compute the *segregation score* as $S_{\textit {ij}}^{\mathrm {S}}\,=\,\!-\log \mathrm {P}_{\textit {ij}}\left (n \leq n_{\textit {ij}}\right)$. These computations are performed analytically and they last few minutes on an ordinary computer even for large systems.

We compute the significance of *N*(*N*−1)/2=703891 potential associations between all pairs of taxa for the observed matrix as well as for 100 random realizations generated through the null model. To correct for multiple testing and reduce the dependence on the number of samples where *i* and *j* are present, scores are transformed into Z scores over random realizations of the null model. (see Additional file 1: Figure S2). Large Z scores are found with the observed matrix but not with realizations.

### Number of aggregations and segregations for comparable significance thresholds

The reconstructed association network depends on the threshold above which associations are considered significant. In order to choose these thresholds in a comparable way for aggregations and segregations, we generate random realizations of presence-absence matrices using the null model *π*_*ia*_, and we treat them in the same way as the observed matrix, computing their null model $\pi ^{\prime }_{\textit {ia}}$, the association scores and the number of inferred associations for given threshold. Since in the null model taxa do not interact, these inferred associations represent false positives. In this way, we estimate the false positive rate (FPR) and the positive predictive value (PPV) as a function of the threshold.

**Figure 1:**
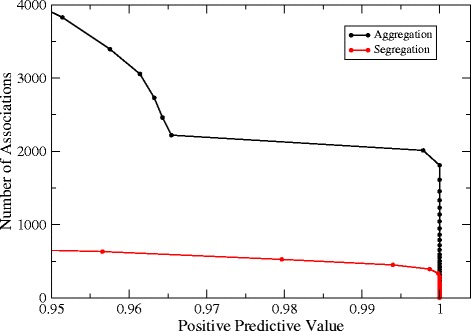
**Number of significant aggregations (black) and segregations (red) found in the observed presence-absence matrix versus the positive predictive value (see** Methods **).**

We plot in Figure 1 the number of inferred associations versus the PPV. For equal PPV, the number of aggregations is larger than the number of segregations. The same qualitative result is found using the FPR as control variable (see Additional file 1: Figure S3).

A possible artefact that can produce this result is that our method does not allow to detect significant associations for all pairs of taxa. For instance, if two taxa never co-occur in the same environmental subtype, they never co-occur in the null model as well and their segregation score is zero. In general, two taxa can have a significant aggregation (segregation) score only if the probability that they always (never) co-occur is smaller than the chosen threshold. To take this into account, for each threshold we consider only pairs of taxa for which both segregation and aggregation can be detected (consensus set). Also in this case aggregation prevails over segregation: for PPV=0.96, which represents a good compromise between completeness and accuracy, we find 2313 aggregations and 628 segregations (see Table 1). The results shown in the following are obtained using these thresholds but considering also pairs for which only one association type can be detected.

**Table 1:** **Properties of networks obtained with Positive Predictive Value** ***0.96***

**Type**	**Pairs**	**Threshold**	**FPR**	**Associations**
Aggregation	All	5.75	4.1·10^−4^	3394
Segregation	All	5.75	2.4·10^−4^	632
Aggregation	Cons.	4.75	2.0·10^−4^	2313
Segregation	Cons.	5.75	6.2·10^−5^	628

We compared our predicted associations with those obtained by Freilich et al. [28]. These authors predicted potential cooperative and competitive interactions of bacterial species based on the simulation of their metabolic networks in different environments, and complemented these associations through the analysis of pairs of species that co-occurr in metagenomic experiments more and less often than expected by chance. This study differs from ours in three important ways: (1) It assesses the significance of aggregations and segregations through the hypergeometric distribution, which only depends on the pair of taxa examined, instead of using a global null model that also accounts for the absence/presence of other taxa; (2) More importantly, it only examines pairs of species that show the same environmental preferences, while our method takes care of removing environmental preferences through the null model (3) Finally, it considers the species level, whereas we detect associations between genera.

Despite these differences, the results are strikingly similar. Out of 62 genera for which we could identify a correspondence with 73 species studied by Freilich et al., we found 26 aggregations and 16 segregations over 1891 possible associations (1.4 % and 0.8%, respectively) and they found 49 aggregations and 16 segregations over 2628 possible associations (1.9% and 0.6%, respectively). Nine of our aggregating taxa were associated with different environments, so that their co-occurrence was not tested by Freilich et al. Of the remaining 17, 11 were co-occurring also in Freilich et al. study (65%). This is a very significant overlap, since the overlap expected by chance is 17×49/2628=0.32. Note that 38 out of 49 pairs co-occurring in their study were not significantly aggregated in ours, presumably because they are associated to the same environment and were filtered out by our null model. None of the segregating pairs coincided in the two studies. We conclude that our method is effective in filtering out pairs that aggregate because of environmental preferences, and that most of the aggregating pairs that it identifies agree with Freilich et al.’s method. On the contrary, segregations do not agree between the two methods (the overlap expected by chance is 0.1, and we find zero), perhaps because they are more difficult to detect.

### Control network

To take into account possible biases caused by our computational procedures, we constructed a control network. We used as the starting point a random presence/absence matrix extracted with the probabilities computed with the null model for each combination of samples and taxa. We computed association scores for all pairs of taxa exactly as for the observed matrix and we assigned associations using thresholds lower than for the observed network (*T*=3.34 instead of 5.75 for aggregation, and *T*=4.60 instead of 5.75 for segregation) in such a way that the number of associations is the same for networks obtained from the two matrices. One should keep in mind that this control network is not a random network, since its construction produces correlations. For instance, since taxa appearing in many samples tend to co-occur with many other taxa, when we decrease the significance threshold they tend to form many aggregations, which produce aggregation propensity (see below).

### Community structure

*Association propensity.* We investigate the community structure of the observed and the control network by measuring the propensity (see Methods) that two taxa associate given that they both associate with a third taxon *k*. This measure is analogous to the clustering coefficient, but it is clearer to interpret since it is negative if the association with *k* disfavors the association between *i* and *j*. There are two types of associations, aggregation (A) and segregation (S), and three conditioning associations: both *i* and *j* aggregate with *k* (AA), both segregate (SS), and one aggregates and the other segregates (AS). We obtain six propensities, which are reported in Table 2. Even the control network generates significant propensities, since taxa present in many samples tend to form many associations and produce positive propensities. However, propensities are much stronger for the observed network, suggesting that they provide non-trivial information on the community structure. The favored triangles are AAA and ASS, whereas it is disfavored that two segregating taxa aggregate with the same taxon (triangle AAS). These patterns are compatible both with the ecological and with the environmental interpretation of aggregations, and they suggest the existence of separate communities such that taxa of the same community aggregate between themselves and segregate from taxa in other communities.

**Table 2:** **Association propensities for the observed and a random network**

**Propensity**	**Observed network**	**Control network**
(A∣AA)	3.59±0.04	1.82±0.10
(S∣AA)	−1.83±0.27	−0.71±0.13
(A∣AS)	−1.91±0.12	−0.42±0.12
(S∣AS)	4.45±0.09	1.32±0.17
(A∣SS)	3.24±0.04	0.42±0.14
(S∣SS)	1.93±0.11	0.62±0.42

(A∣AA) represents propensity of aggregation given two aggregations, and so on (see text).

*Nestednessν* (see Methods) is a property related with the aggregation propensity that has been shown to be enhanced in mutualistic networks [29], although it may also arise from habitat filtering. Strongly nested pairs share many common aggregations, and they are more frequently observed in the observed than in the control network, see Additional file 1: Figure S5. The medians of the two distributions are different at the 1% significance level (Wilcoxon rank sum test).

### Habitat filtering or ecological interactions?

Significant associations may be attributed either to ecological interactions or to habitat preferences. The null model reduces the second possibility by taking habitat preferences into account, since the taxon-specific parameter *p*_*i*_(*A*) vary for each subtype *A* of the environmental classification so that preference for the same subtype would not necessarily result in significant aggregation. Nevertheless, the environmental classification is necessarily coarse, and we cannot exclude that aggregations are due to habitat preferences that the null model fails to reproduce, such as for instance pH, oxygen or light. Disentangling environmental and ecological preferences is very difficult, since interactions in large natural communities of bacteria cannot be directly observed on a large scale. Therefore, in the following we examine indirect evidences that support one or the other interpretation.

### Environmental and phylogenetic relatedness favor aggregation and disfavor segregation

Firstly, we examined the propensity (see Methods) between aggregation and shared habitat preferences. A positive propensity means that pairs of taxa that share the same habitat preference tend to aggregate more often than generic pairs of taxa or, conversely, that aggregated taxa tend to share habitat preferences. This relationship is expected even for a random presence-absence matrix. Therefore, we compared the observed aggregation network with the control network described above.

**Figure 2:**
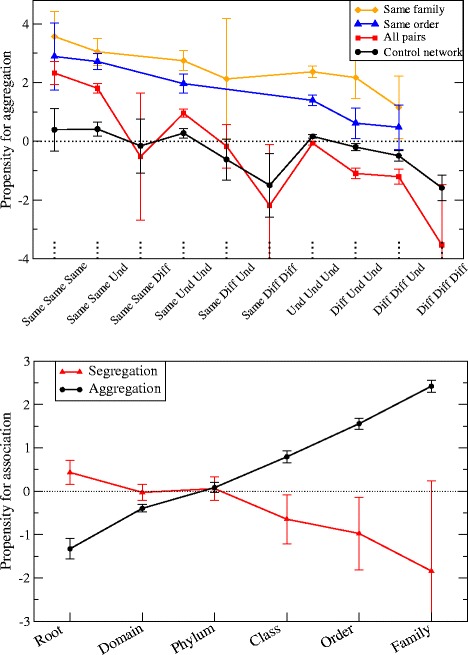
**Propensity to association versus environmental relatedness (top) and versus phylogenetic relatedness (bottom).** In the top figure the x axis labels environmental relatedness at the three levels of subtype, type and supertype. For each level, three values of relatedness are possible: ’Same’ if the preferred habitat is the same, ’Und’ if it is undetermined for one or both taxa, and ’Diff’ if it is different.

Figure 2 (top panel) shows the propensity for aggregation versus the environmental relatedness at the level of subtype, type and supertype. We consider a taxon associated with an environment if more than 50% and at least 3 of the samples in which it is observed belong to this environment. We distinguish three types of environmental relatedness for each level, in decreasing order of similarity: *Same*, if the two taxa are associated with the same environment, *Und*, if one or both of them are not associated with any specific environment, and *Diff* if they are associated with different environments. For instance, (Same, Diff, Und) means that the preference is the same at the supertype level, different at the type level and undefined at the subtype level. We represent in the plot only points for which there are at least 10 pairs, for instance no point is shown for same family and same order and (Same, Same, Diff) habitat preferences.

As expected, one can see from Figure 2 that environmentally related taxa have a strong propensity to aggregate. Nevertheless, in the control network (black curve) the maximum environmental relatedness (same subtype, type and supertype) does not produce significant propensity for aggregation, indicating that the null model is effective in reducing aggregations caused by environmental preferences at the subtype level. At the type and supertype level, small but significant propensities arise even in the control network. Similarly, different supertypes generate a small but negative propensity to aggregate.

These propensities are much stronger for the observed network (red curve) than for the control network, in particular taxa with the same habitat preferences at the supertype level are more prone to aggregate. The most parsimonious interpretation of this observation is that these aggregations are caused by habitat filtering, through environmental preferences that the null model does not take into account. Under this respect, habitat filtering is the preferred explanation for the two points corresponding to share subtype and shared type. Nevertheless, the possibility that some aggregations come from ecological interactions cannot be conclusively rejected either, as discussed in the following sections.

Furthermore, the aggregation propensity is significantly larger for pairs of taxa that are both environmentally and phylogenetically related. This relationship between aggregations and phylogeny goes beyond shared habitat preferences, since pairs of taxa belonging to the same order (blue curve) and family (orange curve) are prone to aggregate even in the absence of a common environmental preference. The propensity for aggregation increases with the phylogenetic relatedness (root, phylum, class, order and family) and the propensity for segregation decreases (see Figure 2, bottom panel), in agreement with the results by Chaffron et al. [17], and also reminiscent of the results by Tamames et al. [26], who found that the environments can be classified based on the affinities that different phyla have with different environments.

The propensity for segregation gives little information, since the number of significant segregations is small. Although we require that significantly segregating taxa co-occur in at least one subtype, we do not find any pair of segregating taxa with the same habitat preference at subtype level, which suggests that the data that we use cannot effectively identify competing taxa. However, most of the segregating pairs that we identify coexist in at least one sample, and the fact that their preferences are different can be also due to the high threshold that we choose to assign habitat preferences.

### Aggregated taxa co-occur in very different environments

To distinguishing habitat filtering from ecological interactions, we envisage two scenarios in which an association due to environmental preferences is not recognized by our null model. The first scenario is that aggregated taxa share a preference for a habitat that occurs in different environmental subtypes, such as nitrite rich habitats found in wastewater treatments and agricultural samples classified in different subtypes, so that the preference is underestimated by the null model. We would expect that this scenario is more likely if the habitat occurs in similar subtypes belonging to the same type (for instance, human gut and mouse gut), rather than in different supertypes (for instance, forest and hydrothermal). The second scenario is that the same sample contains many micro-habitats (for instance, the human gut hosts different environments that cannot be resolved in the most common experimental settings), and the apparent aggregation stems from specialization to different habitats found in the same sample. If this is the case, most of the samples where the taxa co-occur should contain the same micro-habitats, which is more likely if the samples belong to the same subtype or the same type (for instance, human and mouse gut), but not if they come from different supertypes (for instance, open sea and rhizosphere). In both cases, taxa that co-occur in similar samples are more likely to aggregate because of habitat filtering.

**Figure 3:**
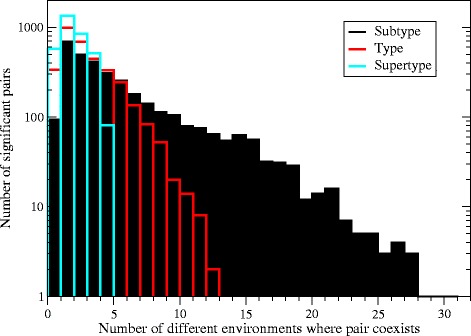
**Distribution of the number of significantly aggregated pairs of taxa that coexist in** ***n*** **different subtypes, types and supertypes.** The number of environments is computed as the exponential of the Shannon entropy. The maximum possible number of environments is 5 at supertype level, 20 at type level and 46 at subtype level.

To test these scenarios, for each pair of significantly aggregated taxa we measured the number of different subtypes, types and supertypes to which the samples where they co-occur belong. This number was measured as the exponential of the Shannon entropy, $-\sum _{i} f_{i}\log f_{i}$, in order to reduce the impact of unfrequent environments. We found that 77% of the significantly aggregated pairs co-occur in samples from more than two different subtypes, 60% from more than two types, and 57% from more than one supertype. These data support the view that most aggregations cannot be explained by habitat preferences. The distribution of the number of different environments shared by each pair of significantly aggregated taxa is shown in Figure 3.

### Cosmopolitan taxa are prone to aggregate

The study of Tamames et al. [26] found that cosmopolitanism, i.e. the fact that some taxa occurr in very diverse environments, is relatively common in the bacterial world, in particular if higher order taxonomic groups are considered. We set up to further investigate the relationship between cosmopolitanism and aggregations because of two reasons: first, since cosmopolitan taxa do not possess environmental specificity, they may allow distinguishing between habitat filtering and ecological interactions; second, this investigation may give hints on whether aggregations play a role in the cosmopolitanism of some bacterial taxa.

We measured taxa cosmopolitanism in two ways: (1) As environmental cosmopolitanism, i.e. the number of different environmental subtypes in which a taxon is present, and (2) As community cosmopolitanism, i.e. the number of different communities in which a taxon is present (see Eq.(1) in Methods). To investigate possible methodological artefacts, we compared the observed aggregation network and the control network.

**Figure 4:**
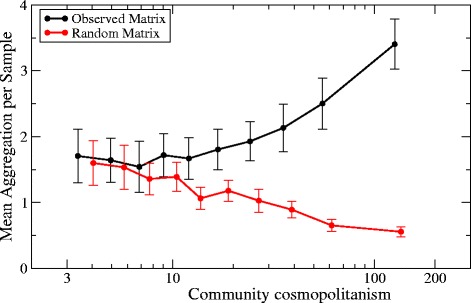
**Number of aggregations of bacterial taxa divided by number of samples in which the taxon is present versus community cosmopolitanism Eq.(** 1 **).** The black lines are obtained for the observed network, the red lines are obtained for a control network. Each point represents the average of all taxa in the same bin of cosmopolitanism.

The number of aggregations of a taxon is positively correlated with its cosmopolitanism both for the control and for the observed network, but in the latter case the correlation is much stronger (*r*=0.64 instead of *r*=0.35). If we normalize the number of aggregations dividing it by the number of samples in which the taxon is present, called prevalence, the relationship with cosmopolitanism remains positive for the observed network whereas it becomes negative for the control network (see Figure 4 for community cosmopolitanism and Additional file 1: Figure S4 for environmental cosmopolitanism). This qualitative difference suggests that the observed relation between aggregations and cosmopolitanism goes beyond the trivial effect that more common taxa are more likely to co-occur. Since cosmopolitan taxa do not present well-defined preferences, it seems unlikely that the excess aggregation is due to habitat filtering. For instance, Flavobacterium and Pseudomonas are present in 36 different subtypes such as Arctic, Mouse Gut, Food Treatment or Mines among others. The hypothesis that their cooccurrence is explained by habitat preferences would imply that these hypothetical preferred properties co-occur in such a wide variety of environments. A more economical hypothesis is that the excess of co-occurrence is explained by cooperative interactions. Another possible hypothesis is that two cosmopolitan have an indirect relationship, due to the fact that there are specialist taxa that, if present, exclude both of them. Our data do not allow distinguishing between direct and indirect relationships, therefore we cannot judge how likely is this hypothesis.

### Known cooperative pairs are found to aggregate

The hypothesis that some of the aggregations that we find are due to cooperative interactions can be tested examining pairs of taxa for which such interactions are known. They fall into three main cathegories: Syntrophy [30], in which one taxon is metabolically dependent on reactions carried out by a different taxon; Biofilms [31], in particular those formed by pathogenic bacteria, which cooperate to promote the chronic nature of the infection [32]; Mutualistic interactions with a shared host [33]. Many pairs of taxa for which there are hints of a cooperative relationship show significant aggregation, as described below.

An important example of syntrophy are methanogenic environments in which organic acids are degraded by syntrophic associations of acetogenic bacteria and methanogenic archaea. Hydrogen consumption by methanogens allows acetogenic bacteria to convert organic acids to acetate and hydrogen [34]. Consistently, we find significant aggregations between Acetobacterium and the methanogen archaea Methanolobus and Methanocalculus. In a similar context, an experimental study of methanogenesis from ethanol identified a three species mutualistic coculture with Desulfovibrio as the ethanol-degrading species producing acetic acid and hydrogen, which was converted to methane by a Methanobacterium sp. while the pH was maintained by the acetate-utilizing Methanosarcina mazei [35]. The two latter taxa show significant aggregation. In another study it was demonstrated that “the coexistence of two types of methanogens, i.e. hydrogenotrophic (Methanoculleus receptaculi) and acetoclastic (Methanosarcina thermophila) methanogens is necessary to respond successfully to perturbation and leads to stable process performance” [36]. These taxa are significantly aggregated. Similarly, the persistence of Pseudomonas putida in an environment with benzyl alcohol as the sole carbon source is dependent on the presence of Acinetobacter. Experimental evolution of this community in a biofilm lead to establish a structured community in which interactions between the two species evolved, enhancing productivity and stability [37]. This is the strongest association that we detect. Nitrosomonas and Nitrospira are two significantly aggregated nitrifying bacteria frequently found in wastewater treatment plants, where they oxidize ammonia and nitrite, respectively [38]. In this case, the aggregation seems to result from habitat preferences and specialization rather than syntrophy.

Another very interesting example of syntrophy are chemolithotrophic bacterial communities that oxidize iron and sulfur leading to the formation of metal-rich acidic water. In these peculiar ecosystems, whose best known example is the acidic river Rio Tinto in Spain, the energetic cycle is characterized by several types of bacteria that act cooperatively [39,40]: sulfur- and iron-oxidizing bacteria, such as Acidithiobacillus ferrooxidans and Leptospirillum ferrooxidans, Acidiphilium, which removes organic compounds toxic for Leptospirillum and reduces iron even in the presence of oxygen, and Acidithiobacillus spp. and members of the Acidimicrobiacea family, which can facultatively reduce iron under anoxic conditions. These taxa have large aggregation scores.

Other interesting examples are related to the ability of bacteria to adapt to environmental conditions that change due to human activity. For instance, Sphingomonas sp. TFEE and Burkholderia sp. MN1, isolated in soils treated with the pesticide fenitrothion, were shown to be able to degrade the pesticide jointly but not alone [41], and they aggregate.

Another important class of examples concern biofilms of pathogenic bacteria. Pseudomonas aeruginosa and Burkholderia, the main pathogens in cystic fibrosis, form mixed biofilms in the lungs of patients. They have frequently exchange genetic material and communicate through a common quorum-sensing system [42]. Their aggregation score is lower than the conservative threshold that we adopt, but it is large (*Z*=3.8). Other associations between pathogenic bacteria are frequently observed in chronic wounds biofilms, in which bacteria cooperate to promote the chronic nature of the infection [32]. The seven taxa most frequently observed in these biofilms break down in two communities, one in which Pseudomonas and Enterobacter are significantly aggregated with Serratia although they are not aggregated between themselves (*Z*=1.5) and another one in which Staphylococcus and Stenotrophomonas are significantly aggregated with Finegoldia and marginally aggregated between themselves (*Z*=4.9), while Peptoniphilus is marginally aggregated with both Finegoldia and Streptococcus.

Finally, an important type of indirect interactions are mutualistic interactions with a common host. Such aggregations can be viewed both as an example of habitat filtering and as an indirect cooperative interaction over evolutionary time scales mediated by the host. Gut and root microbiota constitute the most studied examples and present interesting common features [33]. For instance, Rhizobium tropici and Devosia form a symbiosis with the same aquatic legume host, they have been shown to have interchanged symbiotic genes by horizontal transfer [43] and they are significantly aggregated. Photobacterium and Vibrio, two taxa present in the light organs of some fishs [44], are significantly aggregated.

Summarizing, excluding nitrifying bacteria that are a likely example of habitat filtering, we have examined 26 pairs of experimentally known associations that have different feature that suggest a synergistic relationship, finding that 18 of them (69%) show significant aggregations. Note that two more pairs have large aggregation scores but smaller than our chosen threshold, which indicates that the threshold that we have chosen is strict.

### Network analysis

We now concentrate our attention on a portion of the aggregation and segregation network (the full network of 1187 taxa is too large to be visualized) related with animal Guts, given the major interest on understanding the ecological determinants underlying the assemblage of Human Gut communities. Indeed, it has been suggested that a better understanding of Gut communities may be achieved considering samples from environments different than Human Gut in order to identify the facultative or obligatory nature of the different taxa [4]. With this motivation, we selected a subnetwork containing taxa that has been observed not just in the Human Gut, but also in other guts such as Cattle or Mouse (see Methods). In addition, and for the sake of comparison, we also selected two more subnetworks related with the Saline and Plants environments (see Methods), which are shown in Additional file 1: Figure S8 and briefly commented in Additional file 1: Supplementary text S2.

**Figure 5:**
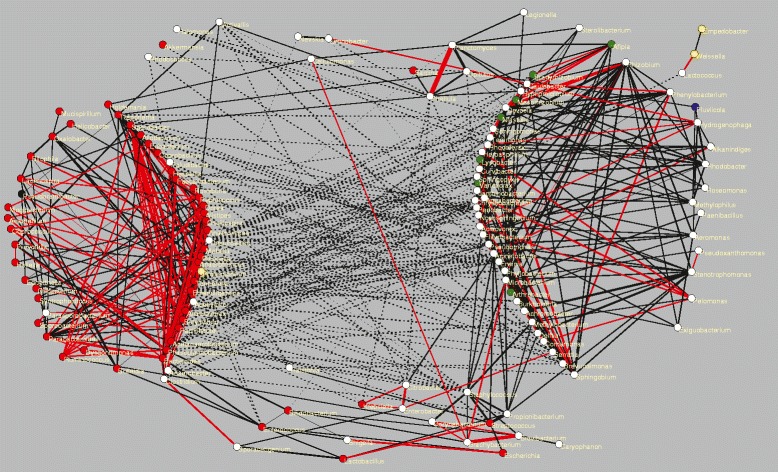
**Networks obtained for taxa present in a group of samples related with animal guts.** Solid lines represent aggregations, dashed lines represent segregations. Circles represent taxa, coloured according to the supertype to which at least 50% of the samples belong. (red = host, green = terrestrial, blue = aquatic, magenta = thermal, yellow = other, white = undefined). Red lines connect taxa belonging to the same family. The graphs have been plotted with the program Pajek [47].

The gut related network shown in Figure 5 comprises 5 subtypes, and we require taxa to be present in at least 3 of them. This condition selects 141 taxa, which are not necessarily preferentially associated with the gut environment. For most of them the association is strong, since 87% are found in at least 5 gut-related samples and 58% in at least 10 samples. Note that, selecting taxa that are observed in at least three Gut subtypes, we underscore ecological relations that may prevail in the Gut independently of the host at the expense of losing some taxa only found in the Human Gut. The 141 selected taxa are related through 468 aggregations and 146 segregations that are computed from the entire data set, so they may co-occur in environments different from the Gut. We can visually distinguish two large groups of strongly aggregated taxa (solid lines) and an intermediate group that links them through transitive aggregations. One of the large groups is constituted by taxa preferentially found in the supertype “host” (red circles), and the other is constituted by generalist taxa (white circles: no supertype accounts for more than 50% of the samples). The two groups are mostly related through segregations (dashed lines) in Figure 5. To quantitatively confirm this structure, we have analysed the modularity of the aggregation network with the modularity algorithm proposed in Ref. [45] implemented in the program Gephi [46]. This algorithm subdivided the Gut related network into five communities: the two large groups clearly seen in the main figure, two intermediate communities connected to both of them and between themselves, and a small community (Enterobacter, Citrobacter and Klebsiella) only connected to the generalist community. They are represented in Additional file 1: Figure S7. There are two possible interpretations of this pattern: associations may be mainly attributed to habitat filtering, which would explain why generalist taxa tend to segregate from gut taxa. Alternatively, associations may be attributed to ecological interactions, and in this case the observed pattern would suggest antagonistic interactions between the gut community and opportunistic invaders.

Finally, we have also compared the network in Figure 5 with independent data obtained sampling the gut microbiota at 5 time points during the first year of life of 13 infants [48]. Interestingly, members of the host-related group appear and remain until the last time point, whereas generalist taxa are intermittently observed at different time points, supporting their interpretation as opportunistic invaders.

## Discussion

Bacterial communities can be very diverse. Hundreds of species have been found in animal gut [49], vagina, mouth, and other organs. However, the global extent of bacterial diversity is currently debated. Estimates based on species-area curves [50] and on the depth of bacterial divisions on the rRNA tree of life [51] anticipated a huge number of bacterial species, but extrapolations from higher taxonomic levels were much lower than expected [52,53]. This discrepancy is due at least in part to the fact that the definition of bacterial species is artificial [54], and very important differences in gene content may exist between individuals classified as the same species, so that the concept of ecotype may be more relevant for bacteria than the rRNA-based species definition [55]. Unfortunately, the resolution of the data does not allow us to address the ecotype level, and we had to conduct this study at the somehow artificial genus level (98% identity in rRNA). It is therefore reassuring that a recent study argued that the bacterial genus level is ecologically coherent [56], supporting the approach undertaken here.

We performed a large scale survey of significant aggregations and segregations of bacterial taxa, adopting a maximum likelihood null model that takes into account environmental preferences at the environmental subtype level. We found a large number of significant aggregations, which may be attributed either to shared habitat preferences that are not taken into account by the null model or to cooperative ecological interactions. Both explanations are at least partially valid. The null model almost eliminates the aggregation propensity of pairs of taxa that share habitat preferences at the subtype level, but not at the type and supertype level (see Figure 2). On the other hand, 18 out of 26 (69%) known examples of cooperative interactions are recovered by our analysis as significant aggregations, and some others have large scores that fall below our chosen threshold, suggesting that the threshold that we use is strict.

In order to quantitatively assess the two kinds of explanation, we examined two main mechanisms that may lead to environmentally driven aggregations: (1) The preferred habitat may be distributed between several environmental subtypes so that the null model does not detect this preference; (2) The same sample may contain several micro-niches, so that the taxa aggregation is only apparent. Both mechanisms are plausible if the aggregated taxa co-occur in very similar environments. Therefore, we conservatively attribute to habitat filtering the aggregation or pairs of taxa with shared environmental preferences. However, we found that most significantly aggregated pairs coexist in more than two different subtypes (77%) and types (60%) and more than one supertype (57%) of the environmental classification, and in these cases habitat filtering appears a less likely explanation of the aggregation.

Cosmopolitanism offers another indirect evidence of the mechanism underlying aggregation. Cosmopolitan taxa, which live in very diverse environmental conditions and communities, present many more aggregations than specialist taxa. The number of aggregations increases with cosmopolitanism faster in the real network than in the network that we use to control methodological artefacts. If shared habitat preferences are the main source of aggregation, we would expect fewer aggregations for cosmopolitan taxa, which lack well defined preferences. Thus this result is consistent with the view that many aggregations are due to ecological interactions.

Cosmopolitanism is apparently at odds with the view that biodiversity is maintained by distinct ecological niches that avoid the competitive exclusion of species. The fact that cosmopolitan taxa tend to aggregate suggests the interesting possibility that cooperative interactions may favor the remarkable cosmopolitanism of some bacterial taxa. Of course, this hypothesis needs to be tested experimentally. We hope that the statistical signal presented here will stimulate such a test. To this end, we provide here examples of groups of cosmopolitan taxa that show strong aggregations between themselves in many diverse environments and can be interesting candidates for experimental studies. (1) The four taxa Pseudomonas, Acinetobacter, Stenotrophomonas and Sphingobium strongly aggregate; interestingly, cooperative interactions between Pseudomonas and Acinetobacter have been observed in experimental evolution [37]. (2) The group of the plant-associated taxa Rhizobium, Arthrobacter, Sphingomonas and Nocardioides, which also co-occur within several scientific papers; (3) The group Devosia, Rhizobium, Lysobacter and Sphingopyxis, the first two associated with plant symbiosis; (4) Streptococcus, Staphylococcus and Propionibacterium, associated with many infectious processes; (4) The aquatic genera Flavobacterium, Acidovorax, Rhodoferax and Polaromonas; (5) The soil bacteria Bradyrhizobium, Rhodoplanes, Conexibacter, Gemmata, Isosphaera and Stella. Some of these taxa, like Nocardioides, Conexibacter, Rhizobium or Byssimonas, are highly promiscuous, forming more than 30 aggregations each.

The aggregation network identified in this work has a marked community structure, in particular it is significantly clustered and nested. The triangles where all three taxa aggregate (AAA) and those where two aggregated taxa segregate from another one (ASS) are statistically favored, suggesting the existence of different communities characterized by intra-community aggregation and inter-community segregation. These data are compatible both with ecological interactions and habitat filtering as the basis of the community structure. Similarly, pairs of aggregated taxa share more common aggregations than expected at random (nestedness). Although habitat filtering can explain this property, it is interesting to note that nestedness is also observed in mutualistic networks of plants and pollinators [29], and it has been suggested to reduce effective competition and favor structural stability and biodiversity [57].

When we compared significant aggregations and segregations, taking care that the comparison is performed at equal false positive rate, we obtained the surprising result that aggregations are more frequent than segregations in the bacterial world. This comparison may be biased by the fact that sparse binary data are little effective at detecting segregation, or that the broad phylogenetic range of bacteria genera makes it difficult to detect competitive exclusion, as it has been recently suggested [58]. Moreover, many of the aggregations that we find must be attributed to habitat filtering. Nevertheless, in most cases this explanation does not appear as the most likely, which opens the way to the surprising ecological interpretation that cooperative interactions between bacteria may be very widespread.

This interpretation is worth investigation. Several other works studied bacterial communities on a large scale, emphasizing the role of either habitat filtering or competitive exclusion or cooperative interactions.

Chaffron et al. [17] performed a study very similar to ours, detecting numerous significant aggregations from bacterial co-occurrence in environmental samples. The main differences with respect to our study are that their null model does not take into account environmental preferences, so that these aggregations must be conservatively attributed to habitat filtering, and segregation was not assessed. The study of Faust et al. examined the microbiome of several body sites with high spatial resolution, finding a comparable number of positive and negative associations within body site [24], but another recent study of the human gut microbiome did not find significant negative associations [23]. Arumugam et al. provided evidence for the existence of three distinct types of composition of the gut microbiome called enterotypes [18]. However, even in distinct enterotypes one does not observe complete exclusion of any common bacterial taxon, except *Prevotella*.

In an interesting study, Freilich et al. studied the ability of pairs of bacterial species to interact competitively or synergistically by predicting their metabolic growth in isolation and in the presence of the other species on different media [28]. Interestingly, in most cases both outcomes are possible depending on the growth medium. They also performed a co-occurrence analysis similar to ours, but with the important difference that it did not adopt any null model and it did not attempt to eliminate pairs that are associated due to common environmental preferences, but instead focused on such pairs. Nevertheless, the qualitative incidence of aggregations and segregations is similar to the one that we found, and 65% of the significantly aggregated genera that we could compare were also co-occurring in their analysis. The co-coccurrence analysis shows that pairs of taxa that are ecologically related through co-occurrence or exclusion tend to have larger competition and cooperation scores than unrelated taxa, which supports the idea that ecological interactions lie behind many aggregation and segregation events. Horner-Devine and coworkers examined 86 matrices of presence-absence of bacterial taxa and computed their C-score [59], finding that all but one significant C-scores were positive, which suggests prevalence of segregation over aggregation [16]. However, the C-score is a global measure that may be positive even in the absence of significant segregations if the majority of pairs co-occur less than expected, which is very likely due to the discretization of presence-absence matrices. Gotelli and Ulrich found that the C-score may be highly significant even if the number of significantly aggregated pairs is larger than the number of significantly segregated pairs [60]. Levy and Borenstein recently studied through metabolic models the complementarity and competition of pairs of bacterial taxa, predicting that taxa that co-occur in the gut microbioma tend to compete more than those that exclude themselves [61]. This prediction suggests that microbiome assembly is dominated by habitat filtering. We also consider habitat filtering as the most economic explanation for the aggregation of taxa that co-occur in one or few environmental subtype, but not for those that co-occur in a wide range of environments. One should be cautious in using metabolic predictions, since the difference between metabolic competition and syntrophy may depend on a small number of key enzymes: The introduction of just one engineered gene in strains of the same bacterial species can turn their competition into a strong synergistic interaction [62]. Moreover, using metabolic predictions it has been shown that it is possible to identify putative media that induce commensalism or mutualism for all the examined pairs of seven bacterial species[63].

There is an increasing number of experiments that attempt to investigate ecological interactions between bacteria on a large scale. A recent experiment measured the overall respiration of assemblies of species and attributed competitive interactions to assemblies in which the total respiration was less than the sum of the respiration of individual species, concluding that competition, not cooperation, dominates interactions among culturable bacteria [64]. However, respiration does not measure biomass production but production plus dissipation, which is expected to increase in the absence of ecological partners [65]. In contrast, another recent experiment found the seemingly opposite result that bacterial taxa have lower growth rate when assayed in the absence of other taxa in their natural community [66], suggesting that cooperative interactions are common. Moreover, a recent experiment found that environmental bacteria are organized into socially cohesive units in which cooperation mediated by antibiotic resistance tends to occur within each ecologically defined population while antibiotic-mediated antagonism occurs between populations [67].

In addition, it is relatively easy to set up experiments in which cooperative interactions evolve or are maintained [68-72], or to find growth media compositions such that the two species are predicted to grow synergistically [28]. A recent work has realized synthetic communities of engineered strains of the same bacterial species linked through the metabolic exchange of amino acids, finding that biosynthetically costly amino acids tend to promote strong cooperative interactions and presenting genomic evidence that suggests that amino acid crossfeeding and synergistic growth are common in bacteria [62].

Last, we discuss the interesting observation that phylogenetically related taxa have large aggregation propensity. This result was also found in Ref. [17,61], where it was attributed to habitat filtering. Nevertheless, this tendency exist also for cosmopolitan bacteria and for pairs that co-occur in many different environments, which suggests that some of these aggregations may be due to cooperative interactions. This hypothesis is puzzling. Since closely related taxa are expected to have large metabolic overlap and to compete strongly, as predicted by the metabolic models of Ref. [61], specialization into different niches or physical separation as in allopatric speciation may be expected to be a likely outcome of a speciation event, leading to segregation between related taxa, which is the contrary of what we observe here. This interpretation is consistent with the recent experiment by Mee et al., who turned strains of the same bacterial species from competitors to cooperators by engineering metabolic dependencies[62].

The recently proposed Black Queen Hypothesis [73] postulates a process in which the evolutionary loss of a gene whose product leaks out of other cells is selectively advantageous for the acceptor strain, which looses the gene and reduces its genome, and neutral for the donor strain, which disposes the gene without additional costs. This model has been proposed as a general mechanism for the establishment of cooperative bacterial communities [74], and its paradigmatic example is thought to be the evolution of genome reduction in several strains of the marine cyanobacteria Prochlorococcus [75].

The observation that phylogenetically related taxa are prone to aggregate may suggest that cooperative interactions played a role in their differentiation. A possible scenario, consistent with the Black Queen Hypothesis and the experiment of Mee et al., who turned strains of the same bacterial species from competitors to cooperators by engineering metabolic dependencies [62], is that one strain lost some genes not needed in its new dominant environment and established an enviroment-dependent metabolic dependency on a sister strain that disposes the products of these genes. This scenario may be testable. In the absence of a direct test, it is just a speculation, and the interpretation that the aggregation between related taxa is mainly due to habitat filtering should be preferred as more economic.

## Conclusions

In conclusion, our results show that aggregations are frequent in the bacterial world, and they occur more frequently for cosmopolitan taxa and for phylogenetically related taxa. Our data support the view that a large number of these aggregations may be due to cooperative interactions. 57% of the aggregations occur in at least two different supertypes, and in our view they are more likely explained by cooperative interactions than habitat filtering, although the latter cannot be ruled out and indirect interactions with a third taxon can offer another possible explanation. Aggregations are particularly common for cosmopolitan taxa that are found in very different environments and for phylogenetically related taxa, which leads us to conjecture that cooperative interactions may be key for the remarkable cosmopolitanism of some bacterial taxa, and they may influence the mechanisms of bacterial differentiation.

## Methods

### Data set

The taxa presence-absence matrix was derived from the data presented in Ref. [26]. Briefly, 3,502 samples of 16S rDNA sequencing experiments were classified into environmental subtypes, types and supertypes and 1187 taxa were identified from the 16S rDNA sequence clustered at 98% sequence identity. Restricted samples, analysed with specific primers with the objective of studying the presence and/or abundance of particular taxa, were identified either from the presence of taxonomic names in the title of the article or identifying samples that contain a single taxon and eliminated from the data set, leaving us with 2322 samples.

### Null model

We implemented the null model proposed in [27], summarized here for completeness. Our data consist of *N* taxa *i*=1…*N* observed at *M* locations *a*=1…*M*, stored in the binary presence-absence matrix *X*_*ia*_∈{0,1}. We want to determine probabilities *π*_*ia*_ that generate random presence-absence matrices $\tilde {X}_{\textit {ia}}$ as similar as possible to the observed one under the assumption that species do not interact and all $\tilde {X}_{\textit {ia}}\in \{0,1\}$ are independent. We assume that there is no preferential association between taxa and locations, an assumption that we will relax later.

We parametrize *π*_*ia*_=*f*(*p*_*i*_*q*_*a*_) so that the probabilities depend on *N* taxon-specific parameters *p*_*i*_ and *M* location-specific parameters *q*_*a*_. Gilpin and Diamond [76] proposed the ansatz *π*_*ia*_=*p*_*i*_*q*_*a*_ and determined *p*_*i*_ and *q*_*a*_ such that the mean of the sum of rows and columns is the same in random matrices as in the observed one. However, as they noted themselves, their model can give probabilities *π*_*ia*_≥1. To avoid this problem, Navarro-Alberto and Manly proposed the ansatz *π*_*ia*_=1− exp(−*p*_*i*_*q*_*a*_), justified assuming Poisson distributed species abundances, and determined the parameters that maximize the likelihood of the observed matrix given the model. The resulting log-likelihood function is $\mathcal {L}= \sum _{\textit {ia}}\left [X_{\textit {ia}}\log (\pi _{\textit {ia}})+(1-X_{\textit {ia}})\log (1-\pi _{\textit {ia}})\right ] \,.$ Maximizing this function, we obtain *N*+*M* equations that we solve with a globally convergent Newton method with analytically computed gradients.

An important drawback of this model is the assumption that taxa do not have habitat preferences. We relax this assumption grouping locations into environmental subtypes and allowing the taxon-specific parameters *p*_*i*_(*A*) to depend on the subtype *A* to which the sample belong. We then solve the maximum likelihood equations separately for samples of each subtype *A*. If taxon *i* is never seen in subtype *A*, then *p*_*i*_(*A*)=0 and *π*_*ia*_=0 for all *a*∈*A*.

### Association scores

The null model allows us to iteratively compute the probability that two taxa *i* and *j* co-occur at *n* locations over *m*, *P*_*ij*_(*n*|*m*):

$$\begin{aligned} P_{ij}(n|m)=&\,P_{ij}(n|m-1)(1-\pi_{im}\pi_{jm})\\ &+P_{ij}(n-1|m-1)(\pi_{im}\pi_{jm}) \end{aligned} $$

This equation, with initial conditions *P*_*ij*_(0|0)=1 and *P*_*ij*_(0|1)=0, yields the probability *P*_*ij*_(*n*|*M*) that the two taxa co-occur at *n* over *M* samples under the null model. We then define the taxon aggregation (TA) and the taxon segregation (TS) scores as

$$\begin{array}{@{}rcl@{}} & & S_{ij}^{\text{TA}}=-\log\left(P_{ij}(n\ge n_{ij}| M)\right) \\ & & S_{ij}^{\text{TS}}=-\log\left(P_{ij}(n\le n_{ij}| M)\right)  \end{array} $$

where *n*_*ij*_ is the observed number of co-occurrences. Sample aggregation (SA) and segregation (SS) are defined in a similar way from the probability that two samples share *n* taxa. These scores are correlated with the number of samples in which individual taxa are present. To eliminate this correlation, we transform them into Z scores as follows. We extract 100 random matrices with the null model of the observed matrix, we compute their null model and, through it, we compute the scores *S*_*ij*_ for all pairs in the random matrix. Finally, we obtain mean and standard deviation of the observed *S*_*ij*_ over the random matrices, and we normalize the observed score subtracting the mean and dividing by the standard deviation.

### Thresholds

In order to choose the significance threshold in an objective way, we estimate the false positive rate FPR (ratio between false positives and total number of pairs), and the positive predictive value PPV (true positives divided by total positives) by generating random association networks with the null model. Namely, we extract a random presence-absence matrix, determine its associated null model and compute aggregation and segregation scores for all pairs. The associations detected in the random network are considered as false positives, and their number is recorded versus the threshold.

### Cosmopolitanism

The environmental cosmopolitanism of a taxon is the number of different environmental subtypes in which it is present, according to the hierarchical classification of Tamames et al. [26]. The community cosmopolitanism is defined as the number of samples in which the taxon is present counting only samples with significantly different communities. We adopt for such a purpose the sample aggregation score *S*^SA^)_*ab*_ that characterizes pairs of samples *ab* that contain more common species than expected by chance, defined similarly as the taxa aggregation score *S*^TA^)_*ij*_. We perform a similar analysis to choose the significance threshold $S_{0}^{\text {SA}}=4.92$ such that the PPV is 0.96. The community cosmopolitanism of a taxon *i* is defined by counting all pairs of samples in which the taxon is present that are below the significance threshold and dividing by all the samples in which the taxon is present:

(1) $$ (\mathrm{Comm.Cosm.})_{i}= 1+\frac{2\sum_{a<b}X_{ia}X_{ib} \vartheta\left(S_{0}^{\text{SA}}-S_{ab}^{\text{SA}}\right)}{\sum_{a}X_{ia}}   $$

The sum in the numerator runs over all pairs of samples where taxon *i* is present, and the theta function selects only significantly different pairs ($S_{\textit {ab}}^{\text {SA}}<S_{0}^{\text {SA}}$). Eq.(1) equals one if all of the communities in which taxon *i* is present are significantly similar, and it equals $m_{i}=\sum _{a}X_{\textit {ia}}$ if they are all different.

### Association between taxa and environments

We associate a taxon with its favored environment at subtype, type or supertype level if more than 50%, and at least 3 of the samples where the taxon is found belong to that environment.

With these criteria, we could assign the dominant environment of 10% of the taxa at subtype level, 30% at type level and 51% at supertype level.

### Propensity

The propensity that two random variables *A* and *B* assume the values *a* and *b* is defined as the logarithm of the ratio between the conditional probability of *a* given *b* and the probability without any condition: Prop(*a*,*b*)= log[P{*A*=*a*|*B*=*b*}/P{*A*=*a*}]= logP{*A*=*a*,*B*=*b*}− logP{*A*=*a*}− logP{*B*=*b*}. The propensity is symmetric exchanging *a* and *b*, it is positive when property *b* favors *a* or the other way round, and negative if the contrary holds.

### Nestedness

In analogy with the definition in [57], we define the nestedness of two nodes *i* and *j* in a network with adjacency matrix *A*_*ij*_ as the fraction of links that they share:

(2) $$ \nu_{ij}= \frac{\sum_{k}A_{ik}A_{jk}}{\sqrt{\sum_{k}A_{ik}\sum_{k}A_{jk}}} \,.   $$

The nestedness is one if *i* and *j* share all of their links, which implies that the clustering coefficient is also one.

## Ethics

The research conducted in this paper did not require ethical approval, since it used previously published data.

## Additional file

Additional file 1 **Supplementary material.** **Figure S1**: Number of samples and number of taxa present in each subtype of the environmental classification of Ref. [26]. **Figure S2**: Distribution of the aggregation and segregation scores for the observed matrix and a random realization. **Figure S3**: Predicted aggregations and segregations as a function of the false positive rate. **Figure S4**: Environmental cosmopolitanism versus the normalized number of aggregations for the observed matrix and for a random matrix. **Figure S5**: Distribution of nestedness between pairs of taxa for the observed matrix and for a random matrix. **Text S1**: describing the clustering of environmental subtypes. **Figure S6**: Hierarchical clustering of the environmental subtypes of Ref. [26]. **Text S2**: Description of networks restricted to subclusters of environmental subtypes related to Plants and Marine. **Figure S7**: Figures for the above networks. **Table S1**: Properties of nets represented in **Figure S8**. **Figure S8**: Propensity to share environmental preferences conditioned to the phylogenetic relatedness. **File Aggregations.txt**: List of 3362 significant aggregations of bacterial taxa and their properties (text file). **File Segregations.txt**: List of 632 significant segregations of bacterial taxa and their properties (text file).
