# Supplementary material for: Bacteria dialog with Santa Rosalia: Are aggregations of cosmopolitan bacteria mainly explained by habitat filtering or by ecological interactions?
Source: BMC Microbiol. 2014 Dec 4;14:284. doi: 10.1186/s12866-014-0284-5 (PMC4263022; doi:10.1186/s12866-014-0284-5)
Supplement: Supplementary file 1 — Supplementary material. Figure S1: Number of samples and number of taxa present in each subtype of the environmental classification of Ref. [26]. Figure S2: Distribution of the aggregation and segregation scores for the observed matrix and a random realization. Figure S3: Predicted aggregations and segregations as a function of the false positive rate. Figure S4: Environmental cosmopolitanism versus the normalized number of aggregations for the observed matrix and for a random matrix. Figure S5: Distribution of nestedness between pairs of taxa for the observed matrix and for a random matrix. Text S1: describing the clustering of environmental subtypes. Figure S6: Hierarchical clustering of the environmental subtypes of Ref. [26]. Text S2: Description of networks restricted to subclusters of environmental subtypes related to Plants and Marine. Figure S7: Figures for the above networks. Table S1: Properties of nets represented in Figure S8. Figure S8: Propensity to share environmental preferences conditioned to the phylogenetic relatedness. File Aggregations.txt: List of 3362 significant aggregations of bacterial taxa and their properties (text file). File Segregations.txt: List of 632 significant segregations of bacterial taxa and their properties (text file). [file 12866_2014_284_MOESM1_ESM.pdf]

# Supplementary Material for the paper: Bacteria dialog with Santa Rosalia: Are aggregations of cosmopolitan bacteria mainly explained by habitat filtering or by ecological interactions?

Alberto Pascual-García, Javier Tamames and Ugo Bastolla

November 6, 2014

## Supplementary figures and tables

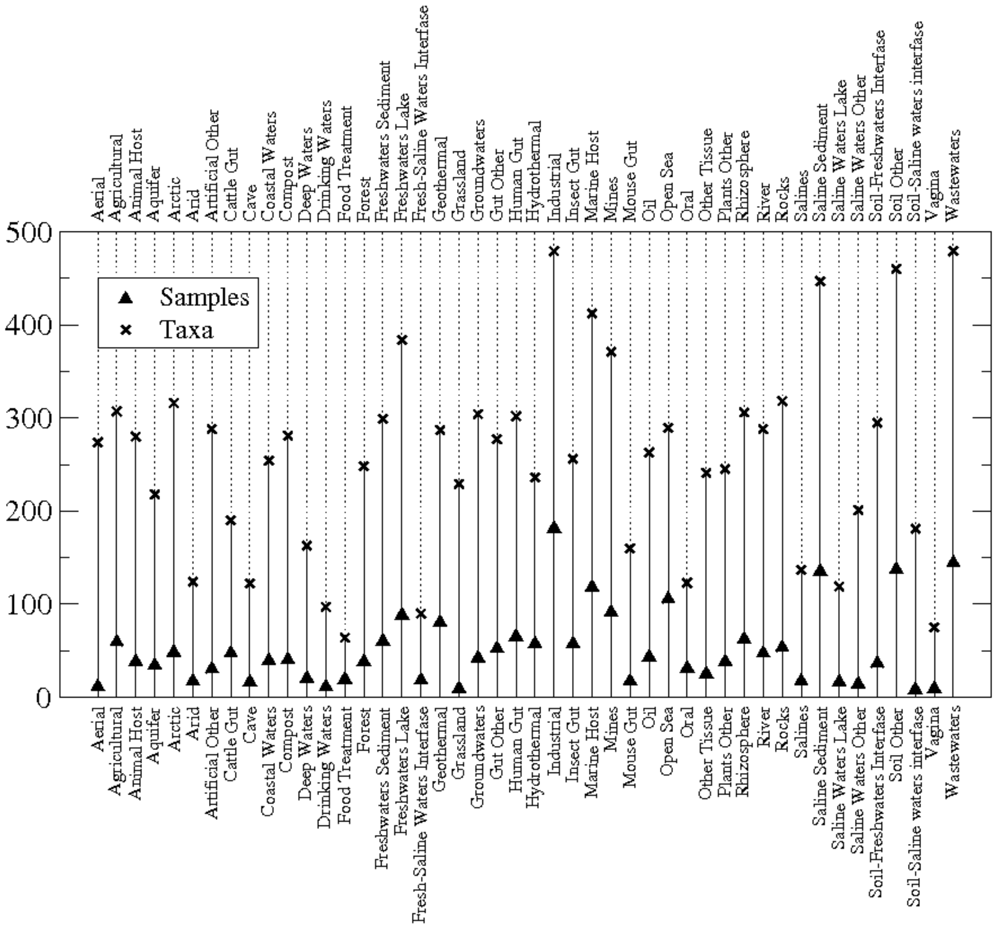


Fig. S 1: Number of samples and number of taxa present in each subtype of the environmental classification of Ref.[1]

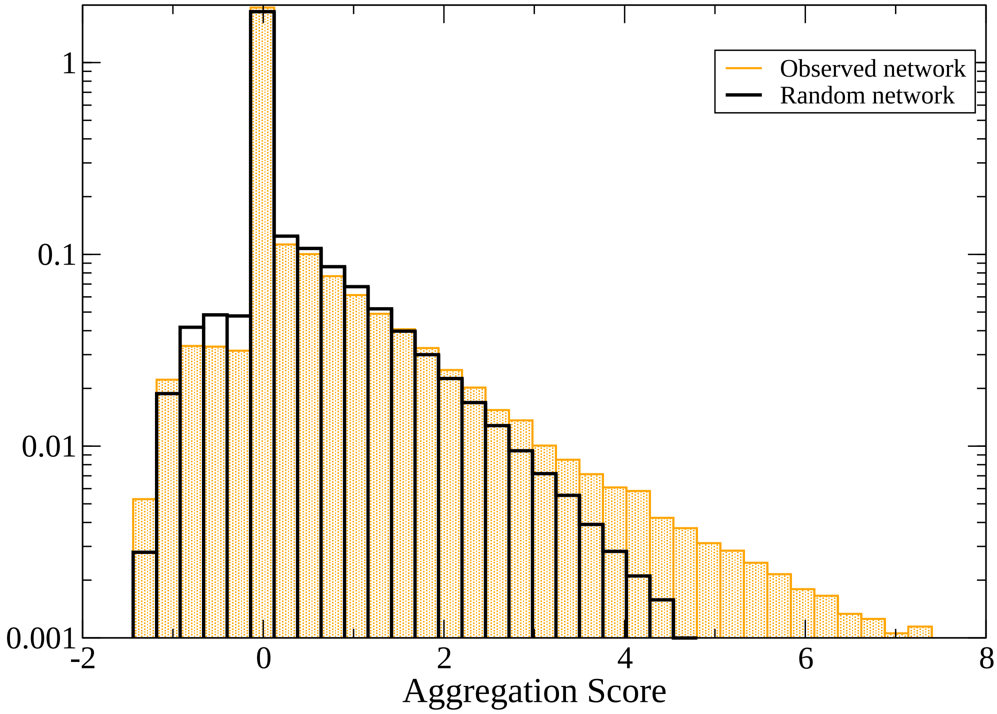


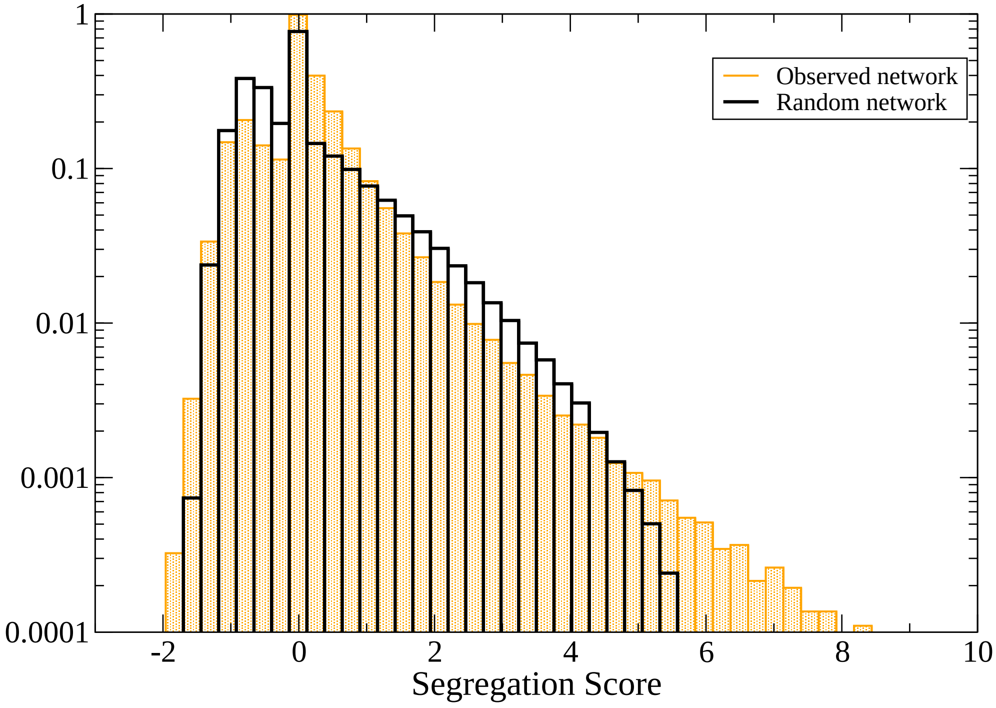


Fig. S2: Distribution of the aggregation and segregation scores for the observed matrix and a random realization. Large scores are found only in the observed matrix, not in the random matrix.

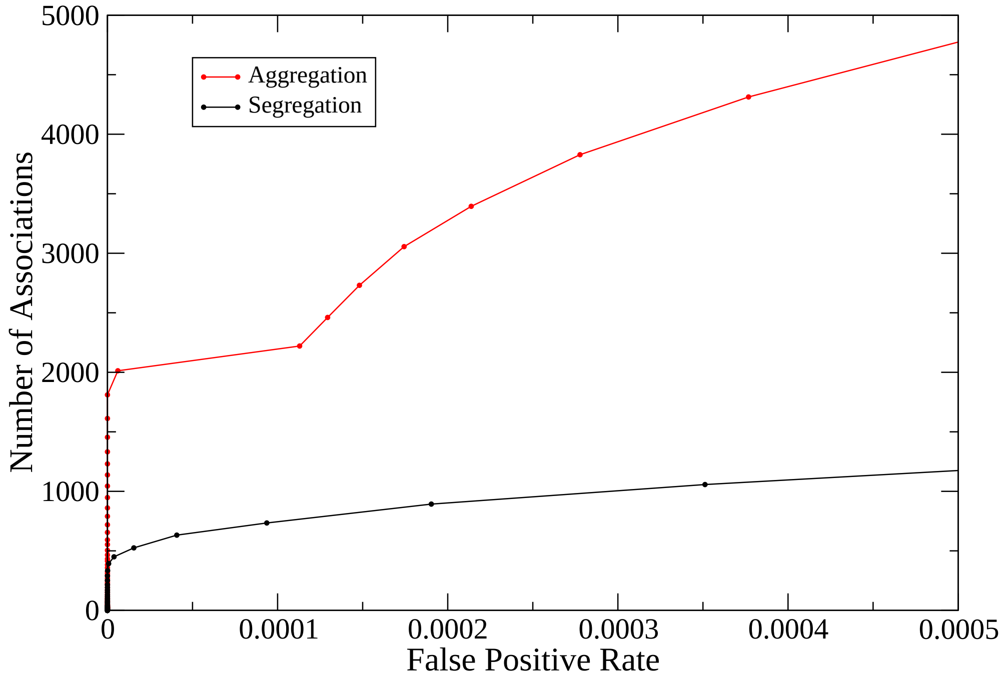


Fig. S3: Number of predicted aggregations and segregations as a function of the false positive rate.

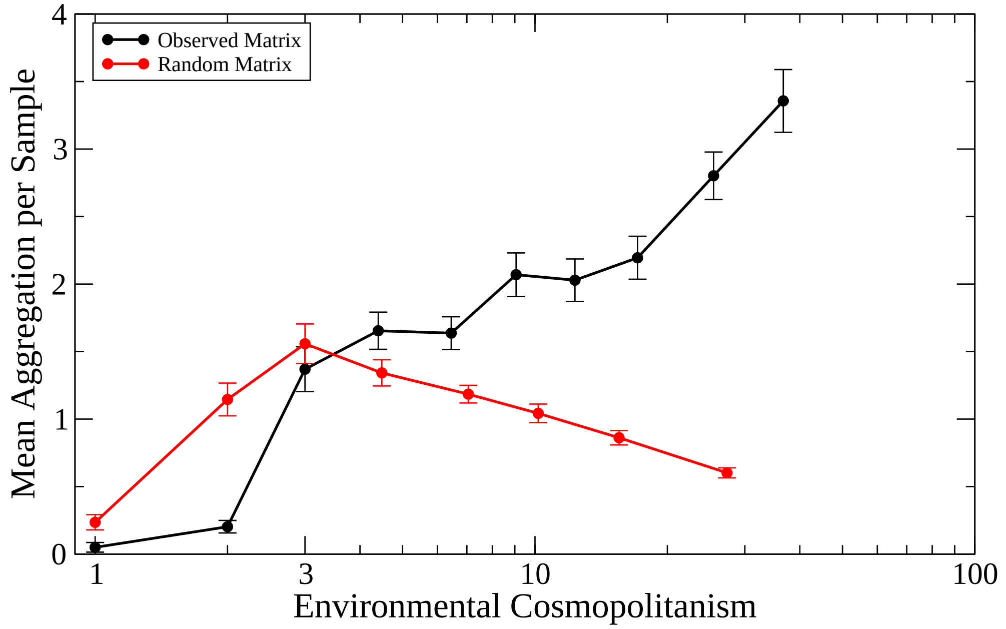


Fig. S 4: Environmental cosmopolitanism, defined as the number of different environmental subtypes in which the taxon is present, versus the normalized number of aggregations for the observed matrix and for a random matrix.

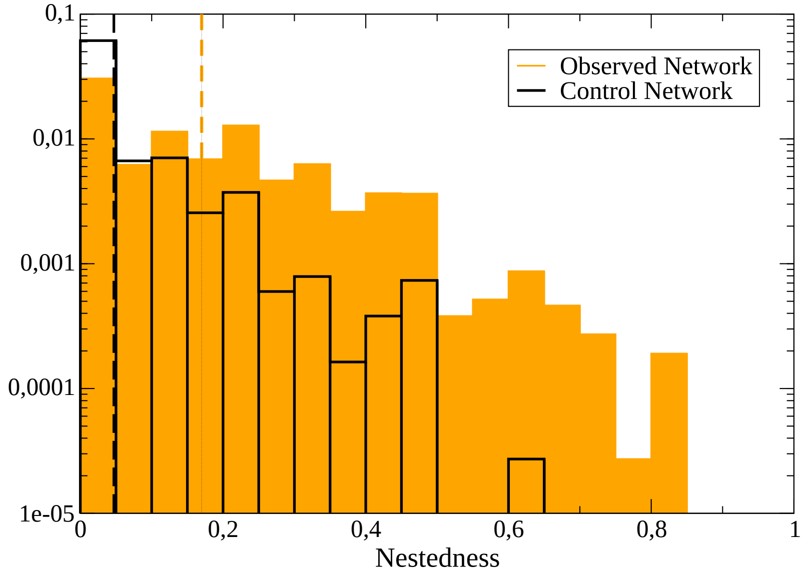


Fig. S5: Distribution of nestedness between pairs of taxa for the observed matrix and for a random matrix.

# Supplementary text S1: Clustering of environments.

We define the similarity between two environmental subtypes measuring how similar are the associations between the taxa observed in these subtypes in the following way. We count the pairs of taxa that associate significantly, either through aggregation or through segregation, and have been observed in both subtypes, and we divide it by the geometric average of the number of pairs observed in each subtype

$$\mathrm{Overlap}(A,B) = \frac{\sum_{i,j} \delta_{ij}^A \delta_{ij}^B L_{ij}}{\sqrt{\sum_{ij} \delta_{ij}^A L_{ij} \sum_{i<j} \delta_{ij}^B L_{ij}}}\,, \qquad (1)$$

where $\delta_{ij}^A = 1$ if taxa $i$ and $j$ have been found in environment $A$ and zero otherwise, and $L_{ij} = 1$ if there is a significant association between $i$ and $j$, zero otherwise. We then transform the overlap into a distance as $D = -\log(\mathrm{Overlap})$, fixing a maximum value $D = 9.99$ if the overlap is zero.

We use this distance matrix to cluster environments by average linkage. The result is shown in Fig.S. For the threshold $d = 3$, we find one singleton and three large clusters, which are related to the types in the environmental classification of Ref.[1]. The second largest cluster contains all the subtypes classified in the supertype host, plus the subtypes aerial (samples coming from exhalations) and compost. The third cluster is related to marine environments and salinity, since it is constituted by subtypes belonging to the types saline waters, saline sediments, hydrothermal and marine host. The subtype food-treatment appears as a singleton that does not belong to any cluster. All other subtypes are contained in the largest automatic cluster (left part of the figure), which contains several interesting subclusters: one gathers all the subtypes related to plants (agricultural, grassland, rhizosphere, forest, plant other), together with arctic and soil other, another subcluster is related to freshwater (subtypes aquifer, groundwater, freshwater sediment, lake, river, soil-freshwater interfase), and it also contains the subtypes geothermal, mines and rocks, the third subcluster is related to industrial activity (industrial, wastewaters, artificial-other and oil), another one is again related to water (saline water lake, freshwater-saline water interfase and, surprisingly, drinking water), finally we find a small subcluster that joins the subtypes arid and cave. These clusters are consistent with the result found in previous work that two of the environmental characteristics that most constribute in shaping bacterial communities are the relation with host and salinity [1]. However, the influence of temperature appears much less relevant, since the subtypes geothermal and hydrothermal are found in very different clusters.

# Supplementary text S2: Representative networks obtained through the clustering procedure

Since the full network of 1187 taxa is too large to be visualized, we represent the bacterial networks present in three groups of similar environmental subtypes with distance threshold $d \approx 1.5$ (see Fig.S).

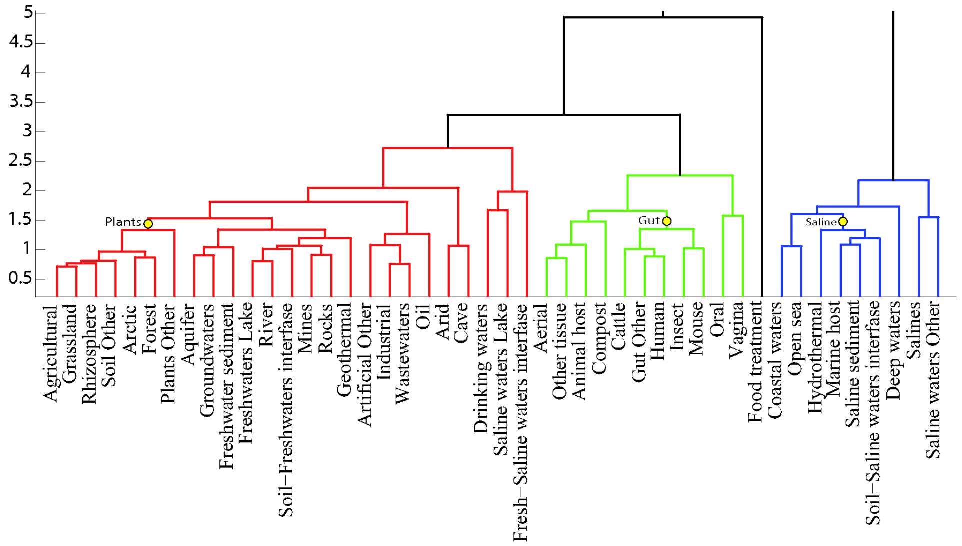


Fig. S6: Hierarchical clustering of the environmental subtypes of Ref.[1] based on the similarity of the bacterial networks that they host (see text).

| Group | Subtypes | $m$ | Taxa | Aggr. | Segr. |
|---|---|---|---|---|---|
| Plants | 7 | 4 | 167 | 716 | 97 |
| Gut | 5 | 3 | 141 | 468 | 146 |
| Marine | 4 | 3 | 189 | 405 | 120 |

Table S1: Properties of nets represented in Fig.S8. For each subcluster, we select only taxa presented in at least $m$ (column 3) subtypes, obtaining a similar number of taxa (column 4).

These groups consist of five subtypes related with the environments Gut (Human, Mouse, Cattle, Insect, Other), seven subtypes related with Plants (Agricultural, Grassland, Rhizosphere, Soil other, Arctic, Forest, Plant other) and four related with marine environments (Hydrothermal, Saline sediment, Marine host, Soil-saline water interfase). For each group of subtypes, we selected those taxa that are observed in at least $m$ subtypes, choosing $m$ in such a way that the number of selected taxa is approximately the same in each group, see Table S1.

Taxa present in Gut related environments are represented in the main text. In Fig.S7 we represent five modules obtained from modular decomposition of the Gut related network with the algorithm of Ref.[2] and implemented in the program Gephi [3]. The first plot represents the first module, prevalently constituted by taxa related with the environment Host. The module in the second plot is prevalently constituted by gen-

eralist taxa. The third plot represents three intermediate modules mainly constituted by host-related taxa.

In Fig.S8 we represent taxa related with Plants and with Marine environments. Significant aggregations are drawn as solid lines, and segregations as dashed lines. These associations are computed from the entire set of samples. One can see that these networks have peculiar properties. The plant related network (top) is connected by a dense net of aggregations, and segregations mostly affect taxa with few aggregations. Most selected taxa have preference for the terrestrial environment. The marine related network (bottom) is less densely connected. Also in this case, we identify two groups characterized by within group aggregations and between group segregation.

# References


[1] J. Tamames, J.J. Abellán, M. Pignatelli, A. Camacho, and A. Moya (2010) Environmental distribution of prokaryotic taxa. *BMC Microbiol.*, 10:85.

[2] Blondel VD, Guillaume JL, Lambiotte R, Lefebvre E (2008) Fast unfolding of communities in large networks J. Stat. Mech. Theor. Exp. 10, 1000

[3] Bastian M, Heymann S, Jacomy M (2009) Gephi: an open source software for exploring and manipulating networks. International AAAI Conference on Weblogs and Social Media.

[4] V. Batagelj, A. Mrvar. Pajek: A Program for Large Network Analysis. Home page: http://vlado.fmf.uni-lj.si/pub/networks/pajek/

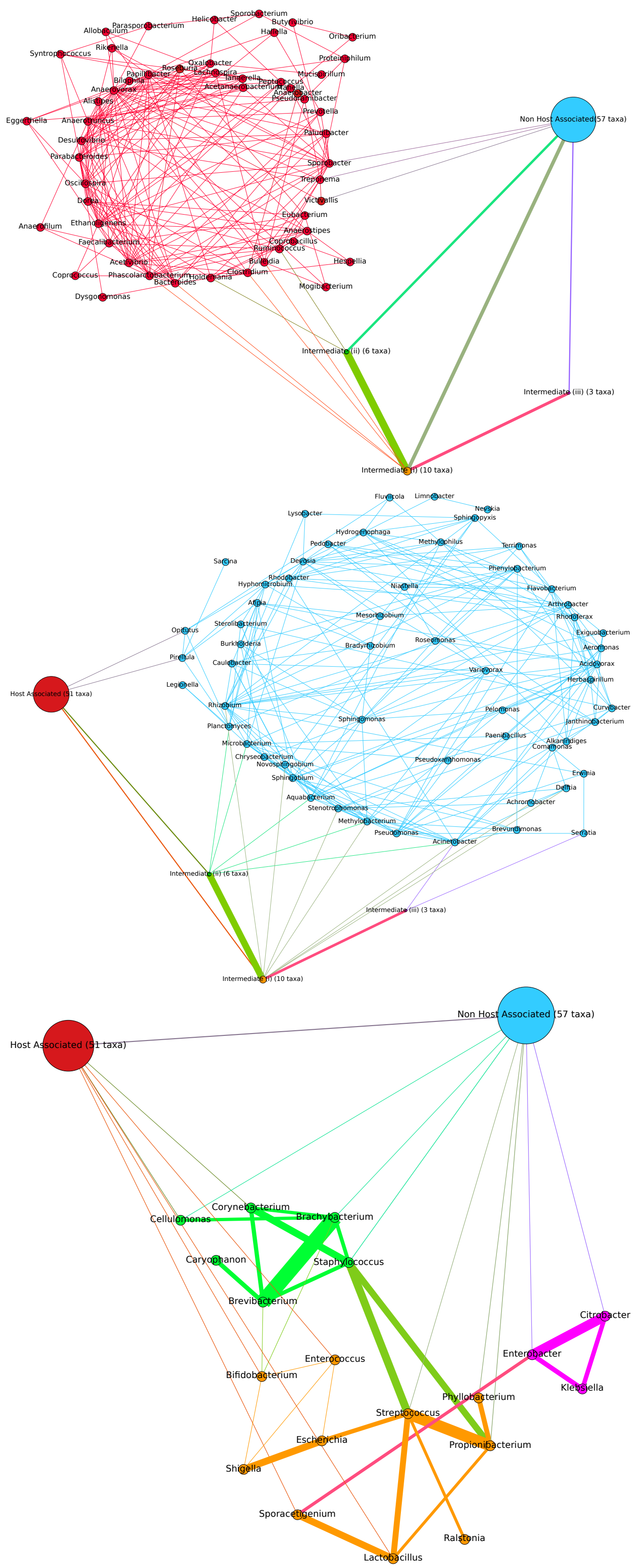


Fig. S7: Networks obtained from modular decomposition of the Gut related network with the algorithm of Ref.[2] and implemented in the program Gephi [3]. The first plot amplifies the first module, prevalently constituted by taxa related with the environment Host. Taxa in the second module are prevalently generalist taxa. The third plot represents three intermediate modules mainly constituted by host-related taxa. For clarity, only aggregations are represented. The graphs have been plotted with the program Gephi [3].

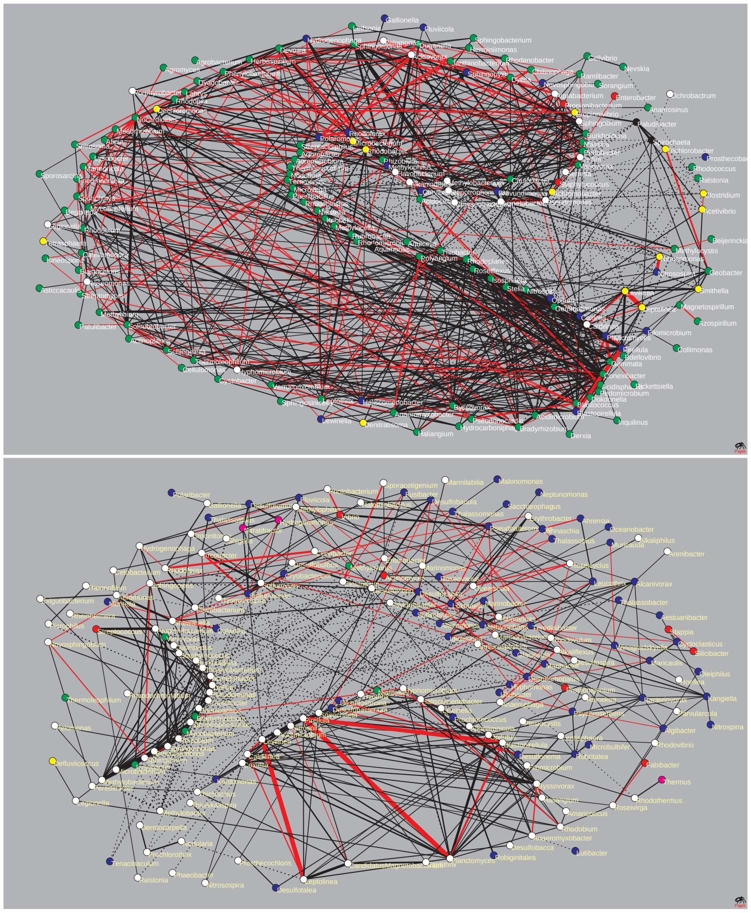

Fig. S 8: Networks obtained for taxa selected in a specific subclusters of environmental subtypes related to Plants (top) and Marine (bottom). Solid lines represent aggregations, dashed lines represent segregations. Circles represent taxa, coloured according to the associated supertype (red=host, green=terrestrial, blue=aquatic, magenta=thermal, yellow=other, white=undefined, black=uncertain). Red lines connect taxa belonging to the same family. The graphs have been plotted with the program Pajek [4]

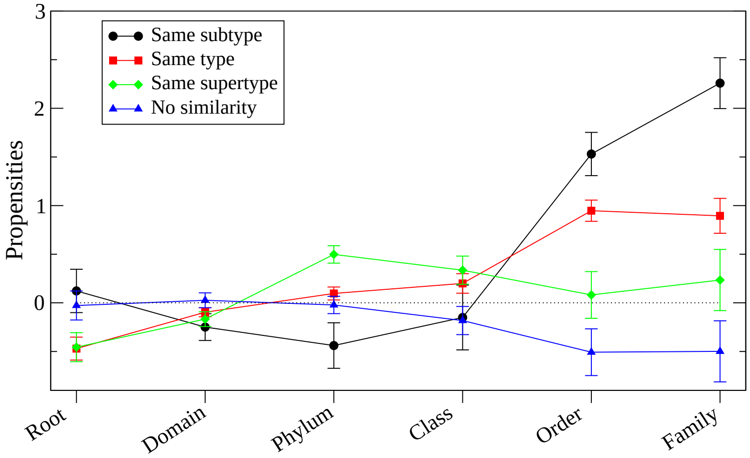


Fig. S9: Propensity to share environmental preferences conditioned to the phylogenetic relatedness.
